# Analysis and Annotation of c‑Type Ions in Peptide Mass Spectral Libraries

**DOI:** 10.1021/acs.jproteome.6c00094

**Published:** 2026-07-14

**Authors:** Meghan C. Burke, Xiaoyu Yang, Yuxue Liang, Pedatsur Neta, Stephen E. Stein

**Affiliations:** Mass Spectrometry Data Center, Biomolecular Measurement Division, 10833 National Institute of Standards and Technology, Gaithersburg, Maryland 20899, United States

**Keywords:** Mass Spectral Library, proteomics, glutamine, asparagine, cysteine

## Abstract

The large collection of high-quality mass spectra in the NIST Human HCD Peptide and NIST Tandem Mass Spectral Libraries is used to understand the role of peptide sequence in c-type ion formation in beam-type collision-induced dissociation. Furthermore, results from the analyses were used to inform and apply annotation rules for c-type ions in the updated Human HCD Peptide Mass Spectral Library. As a result, 155,663 c-type ions are now annotated, providing additional peptide sequence information, often where b- and y-type ions may be lacking. Examples shown here highlight the significance of these c-type ions, both in terms of their relative abundance and in resolving peptide-sequence ambiguities. Furthermore, these examples illustrate the value of including c-type ions to improve searching and scoring of peptide spectrum matches.

## Graphical abstract

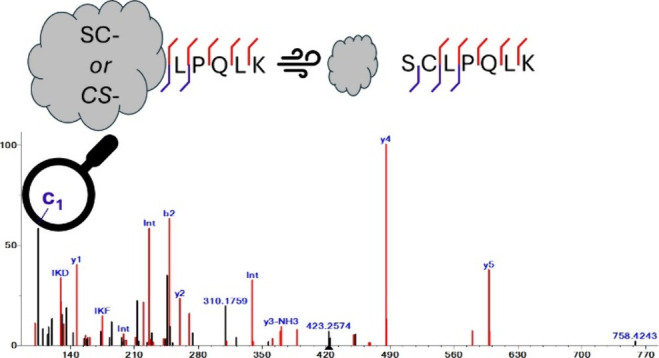

## Introduction

A sequence-based preference for c-type fragment ions, caused by dissociation of the peptide backbone C_α_-N bond, has long been observed in collision-induced dissociation (CID) mass spectra of peptide precursor ions.^1^ The sporadic and selective nature of c-type ions is distinct from what is predominantly observed for b- and y-type ions in CID, which are complementary ion pairs resulting from the dissociation of the peptide amide C–N bond. Importantly, these uncommon sequence ions can provide information where b- and y-type ions are generally in low yield, such as the c_1_ ion.^1–4^ As the b_1_ ion is thought to be unable to form a stable oxazolone structure, the b_1_- and complementary y-type ion are often not observed and, therefore, fragmentation between the first two residues of a peptide sequence is often lacking. Unlike electron transfer dissociation (ETD),^200^ where electrons transferred to protonated peptides generate c- and z-type fragment ions via backbone cleavage at the C_α_-N bond, the unanticipated c-type ions in CID are the result of a multistep fragmentation process.^2^

Investigations into the sequence-based selectivity of c-type fragment ions generated from peptide precursor ions activated by CID and beam-type collision-induced dissociation (HCD) have largely agreed that the residue at the C-terminus of the cleavage site is an important determinant in the formation of the c ion.^1,3,4^ As proteomics aims to tackle more nonmodel organisms and utilize more data independent acquisition (DIA)-based methods, it becomes increasingly important to assign all possible fragment ions, especially in cases where the c_1_ ion can be nearly as abundant as the base peak in a tandem mass spectrum.

The need for c-type ion annotations in the NIST Peptide Mass Spectral Library^5^ became apparent after analysis of NIST Reference Material (RM) 8321,^6^ Peptide Mixture for Proteomics, in which abundant, reproducible, unannotated peaks were visible in the experimental and corresponding reference library^5^ tandem mass spectra. Figure 1 highlights an example of a Human HCD Peptide Library^5^ spectrum for one of the synthetic peptides present in NIST RM 8321,^6^ in which the unannotated peak at *m*/*z* 165.1025 corresponds to the c_1_ ion, protonated phenylalaninamide (C_9_H_13_N_2_O).

**Figure 1:**
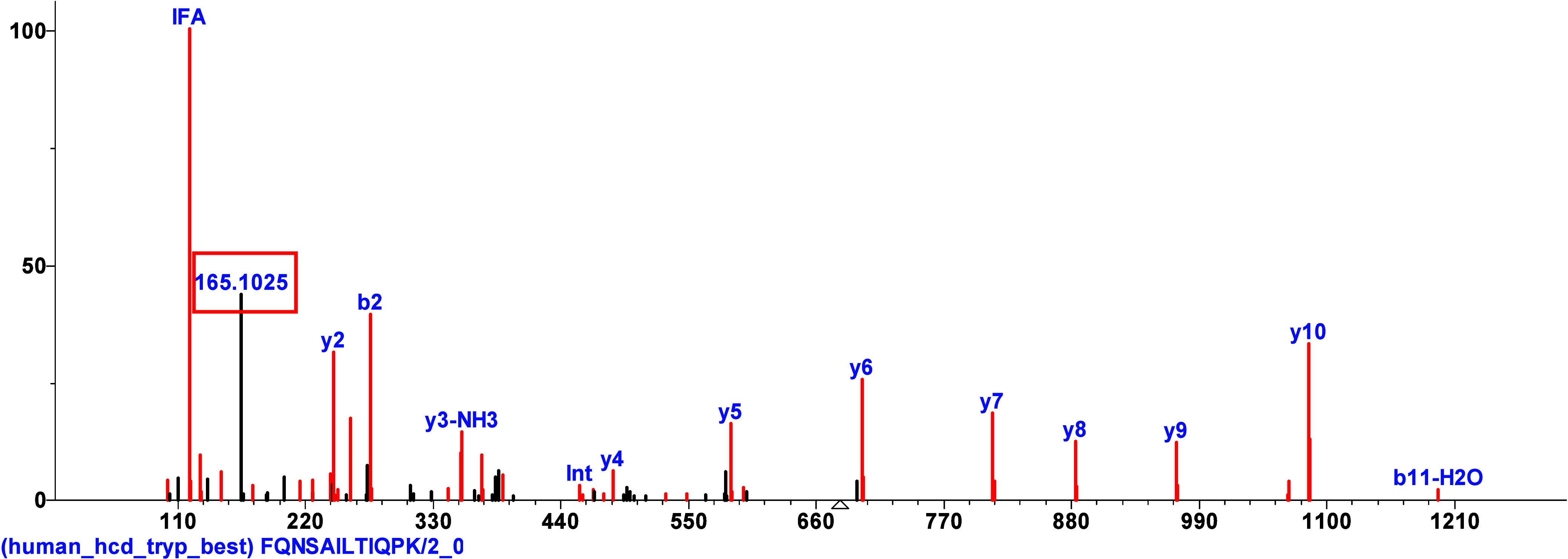
NIST Human HCD Peptide Library spectrum^5^ for peptide sequence FQNSAILTIQPK (*z* = +2), also present in NIST RM 8321.^6^ The unannotated peak at *m*/*z* 165.1025 corresponds to the c_1_ ion, phenylalaninamide. Annotation abbreviations IFA and Int correspond to an immonium ion from phenylalanine (*m*/*z* 120.0811) and an internal fragment ion, respectively.

In this work, the NIST Human HCD Peptide Mass Spectral Library^5^ was used to analyze the occurrence of c_1_ ions in high-quality tandem mass spectra derived from tryptic peptide precursor ions. Furthermore, the extensive collection of tandem mass spectra for dipeptides in the NIST Tandem Mass Spectral Library,^7^ each at several collision energies, was used to better understand the role of the amino acids on either side of the dissociation in the relative abundance of c_1_ ions. Together, these analyses informed annotation rules applied to the updated 2026 NIST Human HCD Peptide Mass Spectral Library.

## Experimental Procedures

### LC–MS/MS Analysis of RM 8321

RM 8321, routinely analyzed as a quality control sample, was chromatographically separated using an UltiMate 3000 (Thermo Fisher Scientific) coupled to an Orbitrap Fusion Lumos (Thermo Fisher Scientific) mass spectrometer. Nanoflow reversed-phase separation was performed on an Acclaim PepMap RSLC 75 μm inner diameter × 25 cm length column (Thermo Fisher Scientific). The flow rate was 300 nL/min with Solvent B, 0.1% (volume fraction) formic acid in acetonitrile, increased as (time (min): % Solvent B) 0:0, 25:2, 142:32, 159:80, 165:98, 175:98, 180:2, and 230:2.

Mass spectra were acquired with the instrument in positive ion mode. The ion transfer tube temperature and spray voltage settings were 275 °C and 1.80 kV, respectively. A scan range of *m*/*z* 380 to *m*/*z* 2000 was chosen for survey scans, or MS1, with a maximum injection time of 50 ms and normalized AGC target of 100%. The RF lens value was 40%. Peptide ions were detected in the Orbitrap with a resolution of 120,000. Filters used in the selection of peptide ions for MS2 included a dynamic exclusion threshold of 15 s, using a precursor tolerance of ± 10 ppm, with isotopes excluded and an intensity threshold of 5 × 10^4^. The total cycle time was 5 s. For both MS^2^ and MS^3^, fragment ions were detected in the Orbitrap at a resolution of 30,000. A maximum injection time of 60 ms was allowed, and the normalized AGC target was 100%. The starting *m*/*z* was fixed to *m*/*z* 70. Lastly, the precursor selection range used for MS^3^ analysis was *m*/*z* 70 to *m*/*z* 250.

### Analysis of c_1_ Ions Present in NIST Human HCD Peptide Library

For c_1_ ion analysis of spectra in the Human HCD Peptide Mass Spectral Library, mass spectra were first required to contain a fragment ion at or below the *m*/*z* of the predicted c_1_ ion (Supporting Information Table S1) to ensure that the c_1_ ion was included in the scan range, if present. The NIST Human HCD Peptide Mass Spectral Library^5^ is divided into three parts based on quality and cleavage specificity, each available as a separate library. The “Best” library contains high-quality mass spectra with no missed cleavages (398,373 mass spectra), while the “Good” library contains medium-quality mass spectra and missed cleavages (200,477 mass spectra). Lastly, the “Semi-tryptic” library contains semitryptic peptides of high and medium quality (312,933 mass spectra). Seventy six percent (302,669 mass spectra) of the Human HCD Tryptic Best Peptide Library met the start *m*/*z* requirement, with those that did not largely starting with glycine (c_1_ *m*/*z* 75.0553) or alanine (c_1_ *m*/*z* 89.0709). NIST MS Search^8^ software was then used to inspect Human HCD Peptide Mass Spectral Library^5^ and search for mass spectra containing peaks corresponding to c_1_ ions. To extract the number of spectra with a given N-terminal amino acid containing a c_1_ ion, the “Any Peaks” search type was used with the predicted c_1_ ion *m*/*z* entered as an “AccurateMZ” peak type using a tolerance of 10 ppm and intensity range of 0–100.

### Analysis of c_1_ Ions Present in the NIST Tandem Mass Spectral Library

Abundance values for c_1_ ions, as well as total ion abundance, were extracted for all dipeptides in the NIST Tandem Mass Spectral Library at all available collision energies. As collision energies can differ between instruments, mass spectra were limited to those acquired on a Thermo Finnegan Elite Orbitrap.

### Annotation of C-Type Ions in NIST Human HCD Peptide Library

To update the NIST Human HCD Peptide Library, the peak annotation software, MS_Piano,^9^ was modified to allow c-type ions if, for position n in a peptide sequence, the amino acid at position *n* + 1 is glutamine, carbamidomethylated cysteine (cysteine­(CAM)), or asparagine. Here, a default mass tolerance of 20 ppm was used.

## Results and Discussion

Analysis of the Human HCD Peptide Mass Spectral Library^5^ (398,373 mass spectra) found that c_1_ ions were present in more than 85% of mass spectra derived from peptide sequences with glutamine or carbamidomethylated cysteine in the second position (Figure 2). Additionally, a c_1_ ion was observed in 30% of mass spectra derived from peptide sequences with asparagine in the second position. The striking preference for c_1_ ions N-terminal to glutamine and, to a lesser extent, asparagine is consistent with previous reports.^2,4,10^ The occurrence N-terminal to cysteine (CAM) has also been noted in the literature.^11^ The scarce reporting of c-type ions N-terminal to cysteine (CAM) may, in part, be due to the requirement of modified cysteine. Importantly, all three amino acids, glutamine, cysteine (CAM), and asparagine, have side chains that contain an amide group (−CH_2_–CH_2_–C­(=O)­NH_2_, −CH_2_–S−CH_2_–C­(=O)­NH_2_, and −CH_2_–C­(=O)­NH_2_ for glutamine, cysteine (CAM), and asparagine, respectively). Therefore, a b_2_ ion containing a C-terminal cysteine (CAM) could also proceed by the mechanisms proposed for that of glutamine and asparagine, via a cyclic imide intermediate, in the formation of the c_1_ ion (Supporting Information Figure S1).^2,4,10^

**Figure 2:**
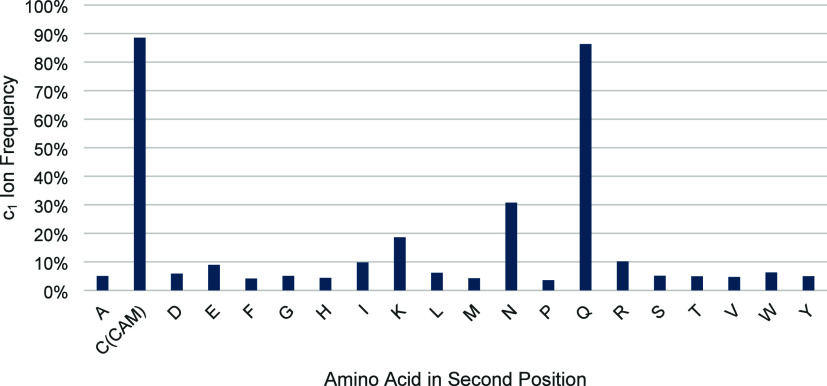
Occurrence of c_1_ ions in the Human HCD Tryptic Best Peptide Library^5^ (398,373 total mass spectra), based on the residue in the second position of the peptide sequence. Here, 302,669 mass spectra were used in calculating the c_1_ ion frequency, as these mass spectra had minimum fragment ions at or below the expected *m*/*z* of the c_1_ ion for that peptide sequence, and cysteine­(CAM) has been abbreviated as C­(CAM).

Next, the collection of synthetic dipeptides in the NIST Tandem Mass Spectral Library^7^ was used to provide further insight into c_1_-ion formation in HCD mass spectra. This collection is a valuable resource as it allows comparison of the fragment ion abundance across several collision energies, and it is reasonable to expect that c_1_ ions may reach maximum relative abundance at different collision energies for different peptide sequences.^12,13^ Breakdown graphs of two dipeptides, Asp-Gln (Figure 3A) and Asp-Glu (Figure 3B), show that the c_1_ ion reaches maximum relative abundance at normalized collision energies (NCEs) of 30 and 40%, respectively. Here, fragment ion abundances are reported relative to the sum of all fragment ions to minimize any bias in ion transmission at different collision energies.^14^ The apex for the c_1_ ion in Asp-Gln (Figure 3A) is 10.39% of total ion abundance, compared to that of 0.18% for Asp-Glu (Figure 3B). In both cases, the c_1_ ion is formed and further dissociates at collision energies similar to those of the y_1_ ion.

**Figure 3:**
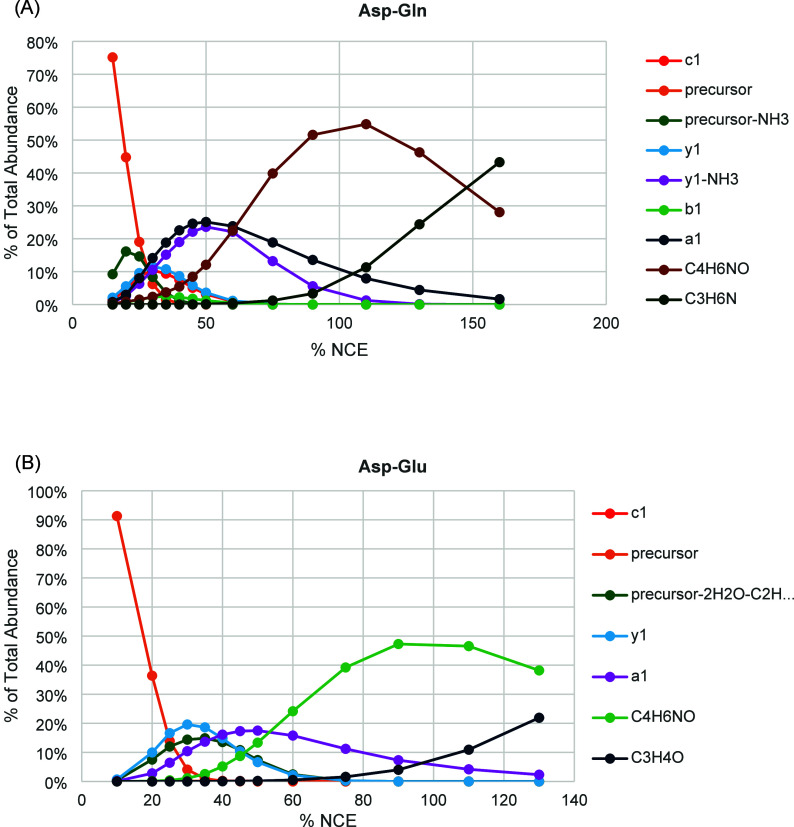
Breakdown plots of dipeptides (A) Asp-Gln and (B) Asp-Glu, where abundance is reported relative to the sum of all product ions at several NCE values.

Given the role of the second amino acid in c_1_ ion formation, the breakdown graphs have been used here to compare the percentage of total ion abundance for c_1_ ions across all dipeptides that share the same N-terminal residue. Comparison of c_1_ ion breakdown graphs for all dipeptides in the NIST Tandem Mass Spectral Library,^7^ for which a c_1_ ion was observed, is included in Supporting Information Figure S2, with selected examples shown in Figure 4. Of note, cysteines in the synthetic dipeptides were not carbamidomethylated and, therefore, c_1_ ions were not observed N-terminal to cysteine. The examples shown in Figure 4 include dipeptides for which the greatest relative abundance was observed for c_1_ ions, Asp-Gln (10.39% relative abundance at an NCE of 30%) and Ser-Gln (8.85% relative abundance at an NCE of 35%). These results, using synthetic dipeptides, confirm the importance of the C-terminal residue in c_1_-ion formation, where the greatest relative abundance was observed in dipeptides with glutamine in this position.

**Figure 4:**
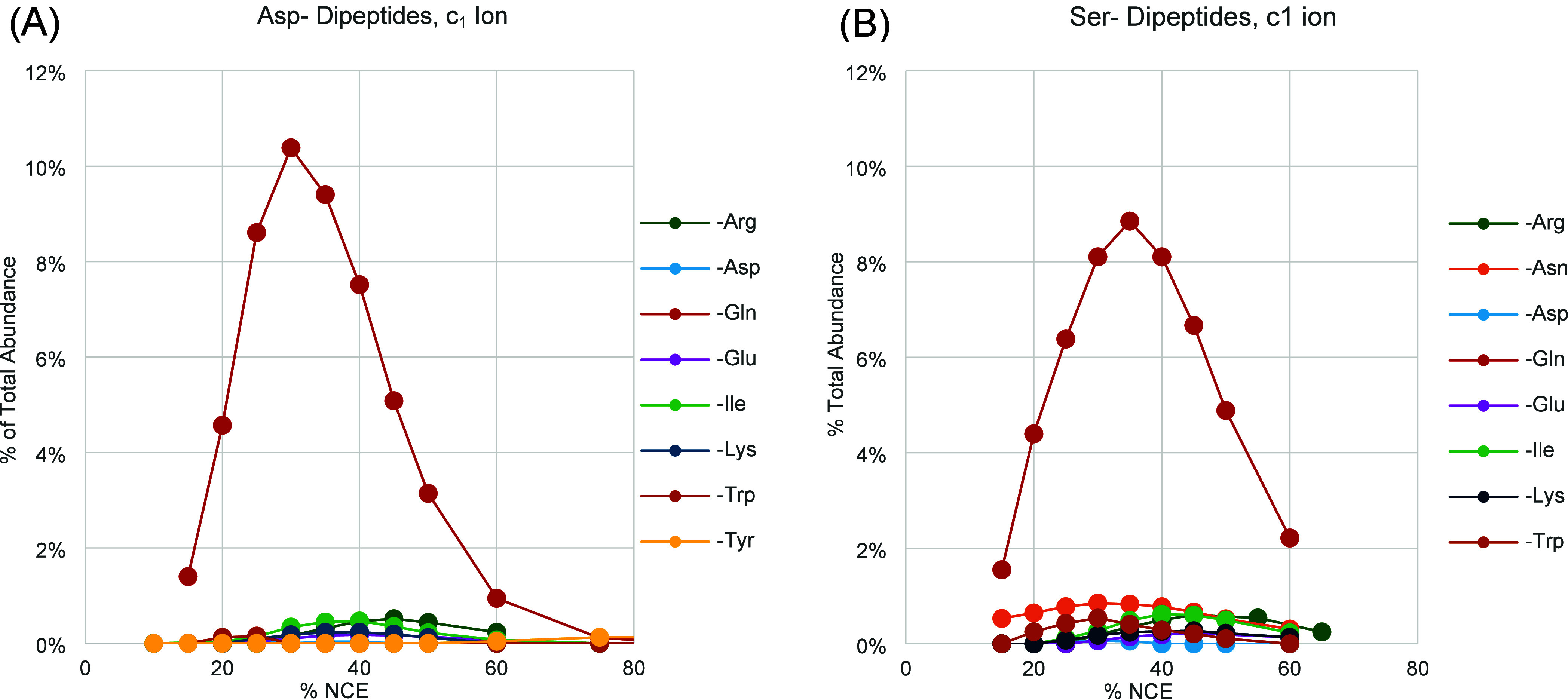
Comparison of c_1_ ion relative abundance for dipeptides in which the N-terminal residue is (A) Asp or (B) Ser. Here, abundance values and total ion abundance were extracted from the NIST Tandem Mass Spectral Library.^7^

A summary of the c_1_ ion relative abundance for all dipeptides in which the C-terminal amino acid is glutamine is shown in Table 1. With the C-terminal amino acid fixed, comparison of dipeptides can provide insight into the role of the N-terminal amino acid in the c_1_ ion relative abundance. As expected, Lys and Arg, which have the largest proton affinity and are capable of sequestering the proton to the side chain, have the lowest c_1_-ion relative abundance. However, linear correlation of c_1_ ion relative abundance with proton affinity is poor (*R* ^2^ = 0.3446, Supporting Information Figure S3). Outliers such as aspartic acid and serine suggest that this may be due, at least in part, to intramolecular hydrogen bonding, which can contribute to gas-phase basicity.^15,16^

**Table 1:** Summary of c_1_ Ion Relative Abundance for All Dipeptides in Which the C-terminal Residue is Glutamine

N-terminal amino acid	max. % total abundance	% total abundance at NCE 30%	proton affinity^17^ (kJ mol^–1^)
Asp	10.39	10.39	905.4
Ser	8.85	8.10	900.4
Ala	7.21	6.92	896.2
Cys	5.60	5.60	895.4
Thr	5.18	4.81	918.4
Asn	3.27	3.27	935.5
His	3.46	2.95	981.1
Phe	2.60	2.60	920.1
Met	1.80	1.80	937.6
Tyr	1.72	1.72	924.2
Val	1.51	1.48	905.8
Ile	1.05	1.05	914.6
Pro	1.20	0.78	929.3
Trp	0.21	0.21	949.3
Lys	0.33	0.15	985.8
Arg	0.09	0.00	1036.8

As positive ion mode is predominantly used in both the Human HCD Peptide Library^5^ and mass spectrometry-based proteomics, the focus here has similarly been limited to c-type ions observed in positive ion mode under CID. However, it is important to note that c-type ions are also observed in mass spectra of dipeptides analyzed in negative ion mode present in the NIST Tandem Mass Spectral Library.^7^ Under negative ion mode, significantly abundant c_1_ ions are observed C-terminal to asparagine, aspartic acid, and phenylalanine.

Lastly, MS^3^ analysis of b_2_ ions generated in the analysis of RM 8321 ^6^ peptides confirmed that the c_1_ ion is a dissociation product of the b_2_ ion for sequences in which a c_1_ ion is expected to occur, N-terminal to glutamine and asparagine. An example is shown in Figure 5. Taken together, the results of RM 8321 ^6^ and NIST Tandem Mass Spectral Library^7^ informed new fragment ion annotation rules now added to the Human HCD Peptide Mass Spectral Library.^5^ As the c_1_ ions discussed here are the result of multistep fragmentation, annotation of c-type ions has been extended to longer c-type ions because the instability of longer b-type ions, relative to their y-type counterpart, can similarly produce these unexpected fragment ions.^10,18^ The updated library includes c-type ion annotations where, for *m*/*z* corresponding to c_n_ ion, the amino acid at n+1 must be glutamine, cysteine (CAM), or asparagine. As a result, 155,663 c-type ions are now annotated, with a summary across c_n_ ion types provided in Table 2.

**Figure 5:**
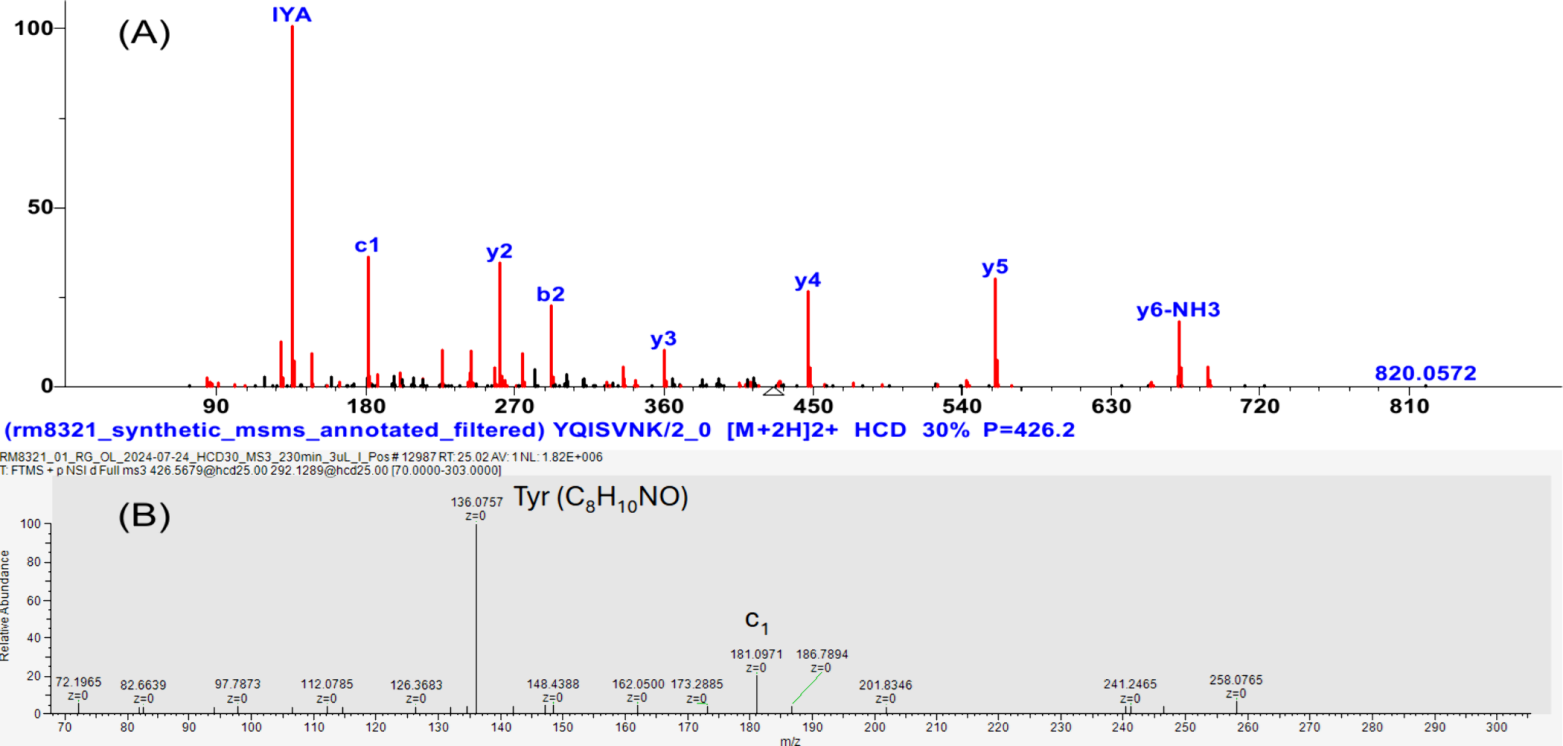
(A) Annotated tandem mass spectrum for the RM 8321 precursor *m*/*z* 426.2 assigned to the peptide sequence YQISVNK (*z* = +2). The annotation abbreviation IYA corresponds to the immonium ion from tyrosine (*m*/*z* 136.0758). (B) MS^3^ analysis of b_2_ ion (*m*/*z* 292.1291) for peptide sequence YQISVNK (*z* = +2) with a c_1_ ion present at *m*/*z* 181.0971 (predicted *m*/*z* 181.0972).

**Table 2:** Summary of Newly Annotated C-Type Ions in the Human HCD Tryptic Peptide Library^a^

c_n_ion type	# annotated peaks in best library	# annotated peaks in good library	# annotated peaks in semitryptic library
1	11898	6222	9791
2	11164	4256	9652
3	15840	5992	12117
4	10459	4146	7527
5	7007	3111	4845
6	5232	2261	3656
7	3298	1457	2567
8	2089	904	1807
9	1335	563	1171
10	868	387	764
11	529	248	458
12	365	148	332
13	220	99	161
14	128	68	118
15	78	40	58
16	55	17	41
17	36	10	17
18	16	4	13
19	10	6	2

a The library is available in three parts, and the now annotated c_n_ ions are summarized for each part, which include best (high quality, no missed cleavages), good (medium quality, includes missed cleavages), and semi-tryptic (high and medium quality, mostly semi-tryptic)

As an illustration of the benefit of peptide N-terminal sequencing, made possible with the use of c_1_ ions, Figure 6 includes a Human HCD Peptide Library mass spectrum,^5^ where a fragment ion corresponding to c_1_ ion corrected an error in the order of the first two amino acids of the assigned peptide sequence. In this example, the assigned peptide sequence C­(Carbamidomethyl)­SLPQLK to the mass spectrum resulted in an unannotated fragment ion at *m*/*z* 105.0663 (58% of base peak). This ion corresponds to the c_1_ ion from Ser. Moreover, our analysis has shown that carbamidomethylated cysteine selectively forms a c-type ion for the amino acid N-terminal to it in a peptide sequence. Therefore, an abundant c_1_ ion is expected when the newly proposed sequence of SC­(Carbamidomethyl)­LPQLK is assigned to the mass spectrum shown. Importantly, as there is no b_1_ ion for the sequence shown, the use of the c_1_ ion is the only way to distinguish between the two peptide sequences, each of which can be assigned to a human protein in UniProt.^19^

**Figure 6:**
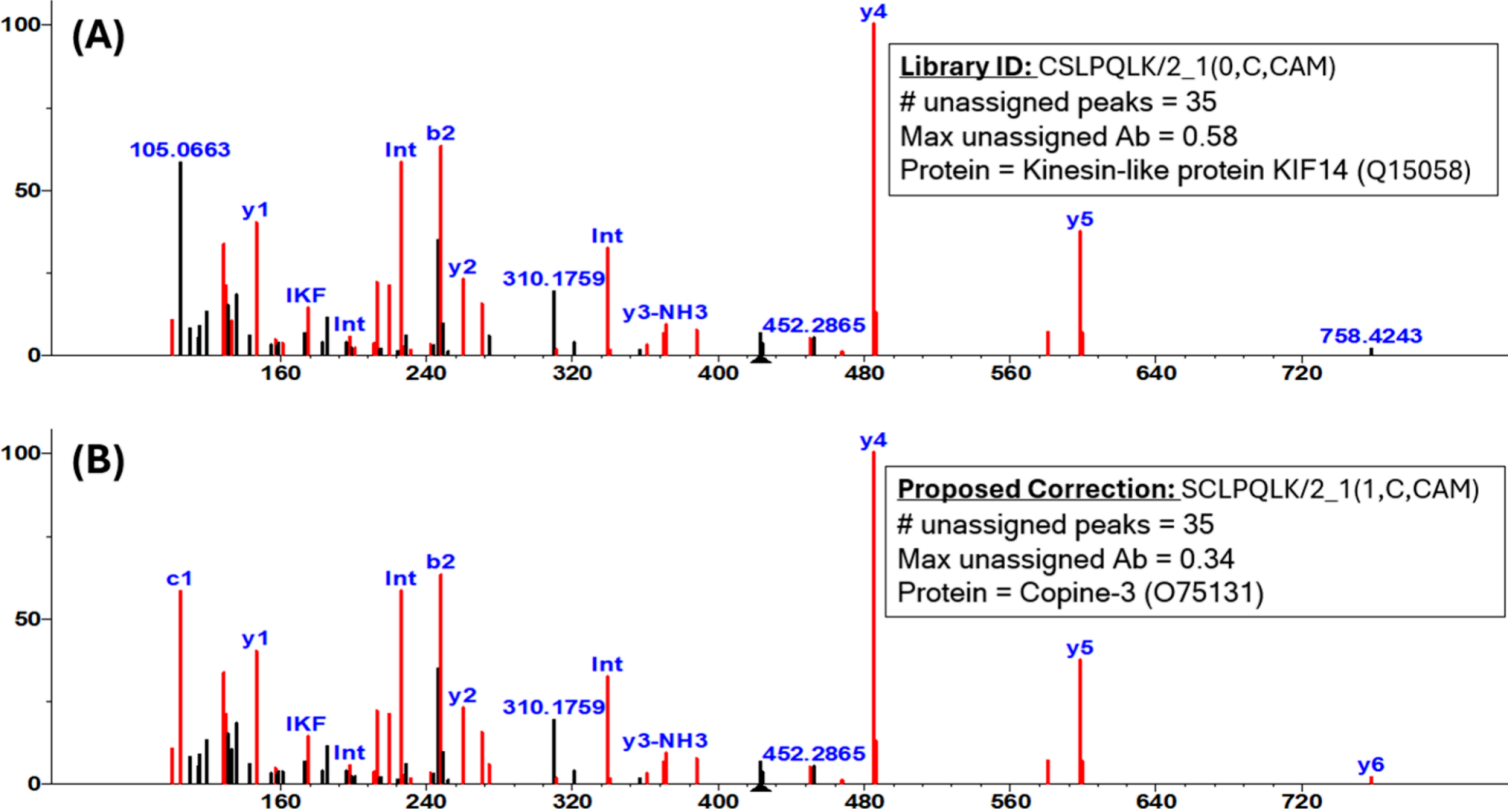
(A) NIST HCD Human Peptide Library mass spectrum^5^ assigned to peptide sequence C­(Carbamidomethyl)­SLPQLK (*z* = +2) and (B) proposed correction based on the abundant unannotated peak corresponding to c_1_ if the order of the first two amino acids is switched. Annotation abbreviations shown as IKF and Int correspond to the immonium ion from lysine (*m*/*z* 175.1191) and internal fragments, respectively.

## Conclusions

The annotation of uncommon c-type ions in the NIST Human HCD Peptide Library presented here expands the level of peptide sequence information available in the NIST Human HCD Peptide Library. The knowledge gained from c-type ions can be applied to both data acquisition and data analysis. For the latter, these now annotated peaks call attention to the value in considering *all* fragment ions, common and uncommon, produced by a peptide ion in assigning and scoring peptide spectrum matches—a major advantage of mass spectral library searching. The results presented here can also inform the *m*/*z* range selected for proteomics experiments, as the predicted *m*/*z* for a c_1_ ion produced by glycine, the smallest amino acid, is *m*/*z* 75.0553 and may fall below the starting *m*/*z* commonly chosen.

## Supplementary Material



## Notes

Supporting Information Figure S1: mechanism proposed by Grzetic and Oomens^11^ for the formation of c_1_ ions from a peptide ion containing glutamine in the second position; Supporting Information Figure S2: comparison of c_1_ ion relative abundance; Supporting Information Figure S3: correlation between c1 ion relative abundance and proton affinity; and Supporting Information Table S1: Chemical formula and corresponding monoisotopic *m/z* values for c_1_ ions for each amino acid (PDF)
