# Supplementary material for: Analysis and Annotation of c‑Type Ions in Peptide Mass Spectral Libraries
Source: J Proteome Res. 2026 Jul 14;25(8):4315–21. doi: 10.1021/acs.jproteome.6c00094 (PMC13459556; doi:10.1021/acs.jproteome.6c00094)
Supplement: Supplementary file 1 [file pr6c00094_si_002.pdf]

Supporting Information

Analysis and Annotation of c-type Ions in Peptide Mass Spectral Libraries

Meghan C. Burke[1]*, Xiaoyu Yang[1], Yuxue Liang[1], Pedatsur Neta[1], Stephen E. Stein[1]

*Meghan C. Burke: meghan.burke@nist.gov

[1]Mass Spectrometry Data Center, Biomolecular Measurement Division, National Institute of Standards and Technology, Gaithersburg, MD 20899

Table of Contents:

1. Supporting Information Figure S1. (A) Mechanism proposed by Grzetic and Oomens[1] for the formation of c_1 ions from a peptide ion containing glutamine in the second position. Here, attack from the side chain amide nitrogen yields a cyclic imide. (B) Dissociation between the N and glutarimide yields a c1 ion.
2. Supporting Information Figure S2. Comparison of c_1 ion relative abundance, where each pane contains dipeptides that share the same N-terminal amino acid. Here, abundance values and total ion abundance were extracted from the NIST Tandem mass spectral library.
3. Supporting Information Figure S3. Correlation between c1 ion relative abundance and proton affinity, for which the values are listed in Table 1.
4. Supporting Information Table S1. Chemical formula and corresponding monoisotopic m/z values for c_1 ions for each amino acid.

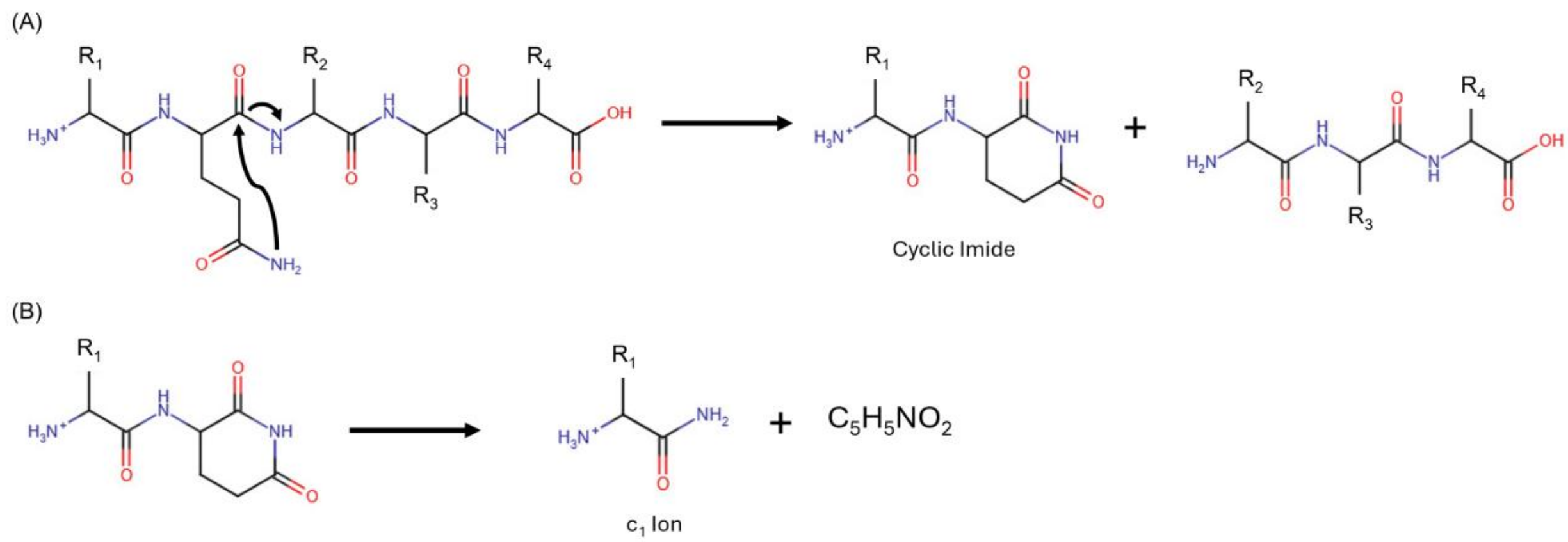

Supporting Information Figure S1. (A) Mechanism proposed by Grzetic and Oomens[1], following proton migration, for the formation of c_1 ions from a peptide ion containing glutamine in the second position. Here, attack from the side chain amide nitrogen yields a cyclic imide. (B) Dissociation between the N and glutarimide yields a c1 ion.

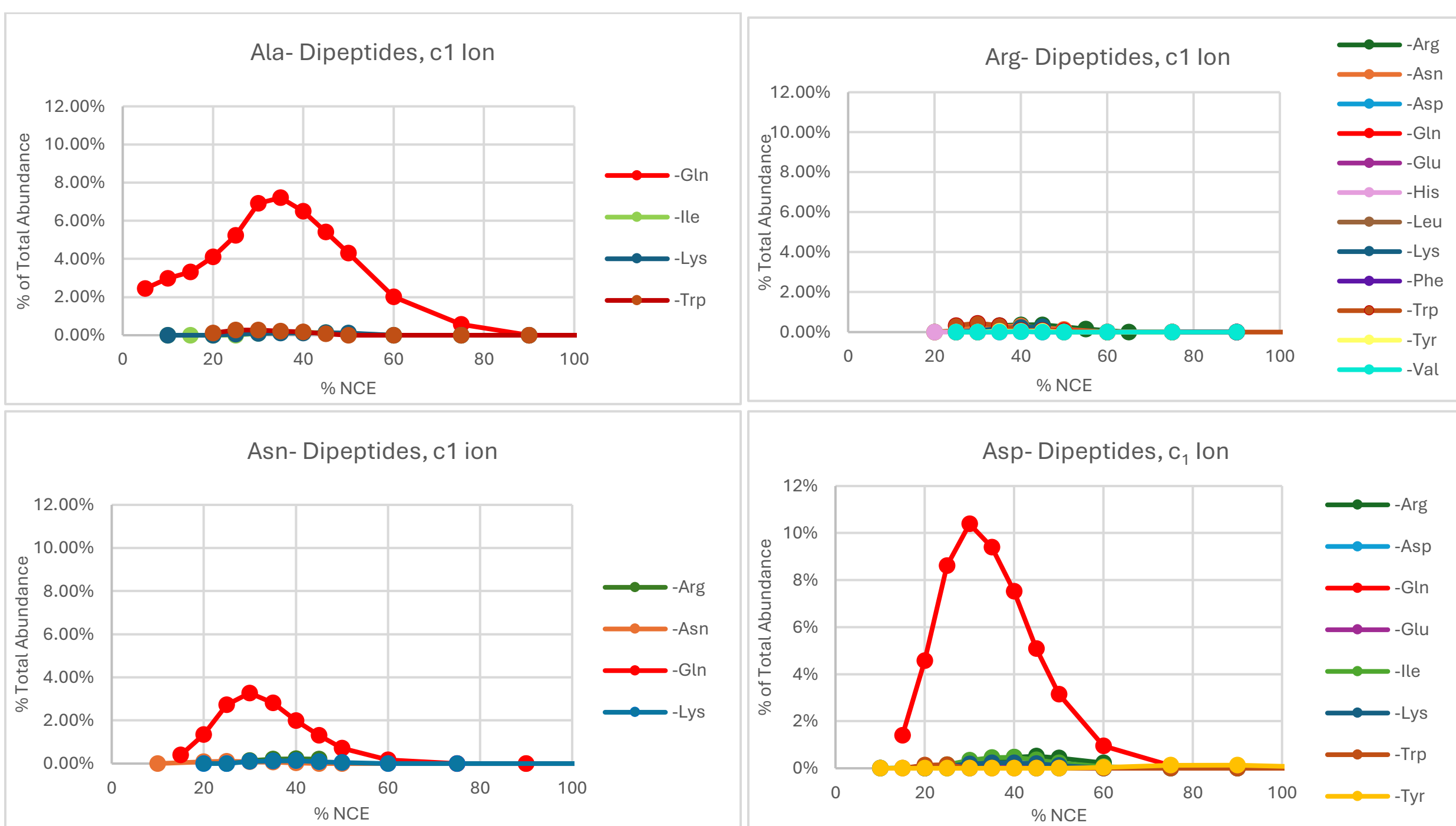

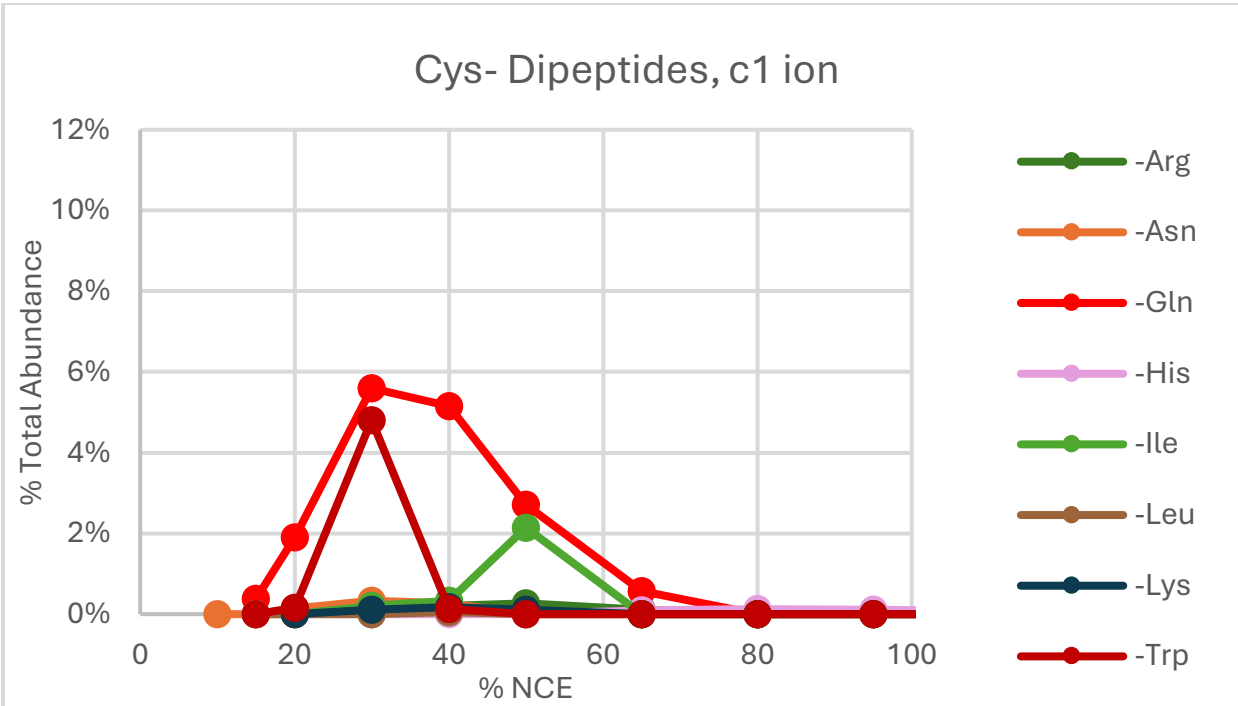
Cys- Dipeptides, c1 ion
% Total Abundance
12%
10%
8%
6%
4%
2%
0%
0
20
40
60
80
100
% NCE
-Arg
-Asn
-Gln
-His
-Ile
-Leu
-Lys
-Trp

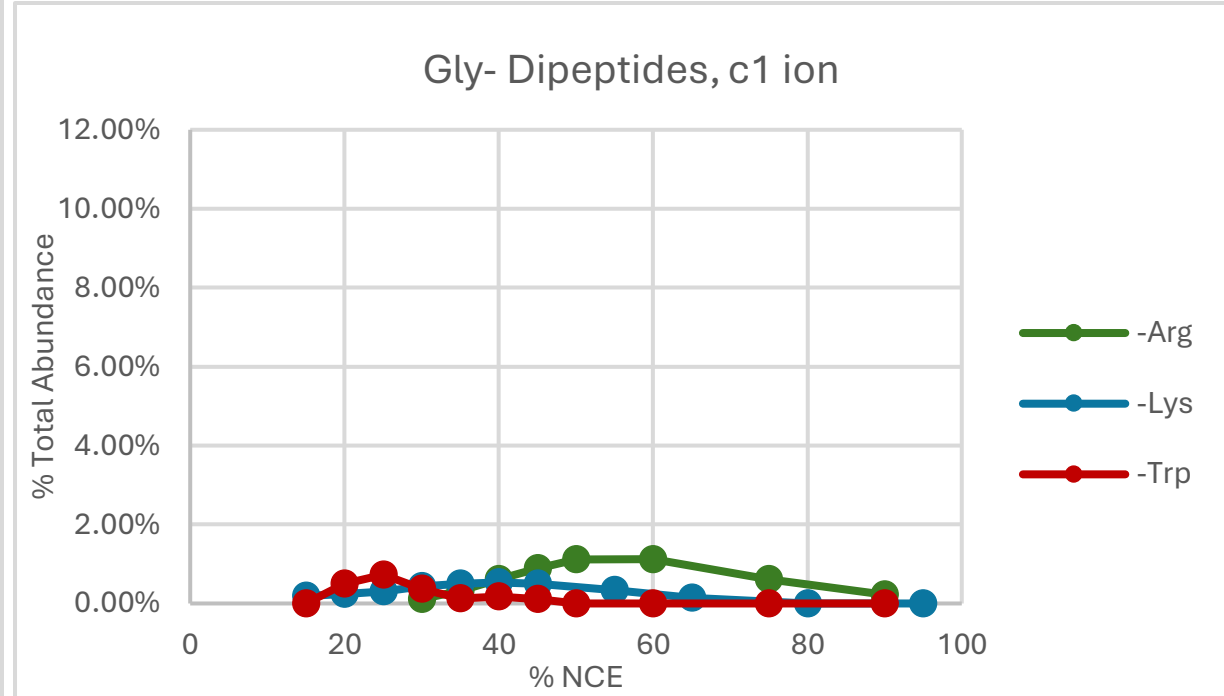
Gly- Dipeptides, c1 ion
% Total Abundance
12.00%
10.00%
8.00%
6.00%
4.00%
2.00%
0.00%
0
20
40
60
80
100
% NCE
-Arg
-Lys
-Trp

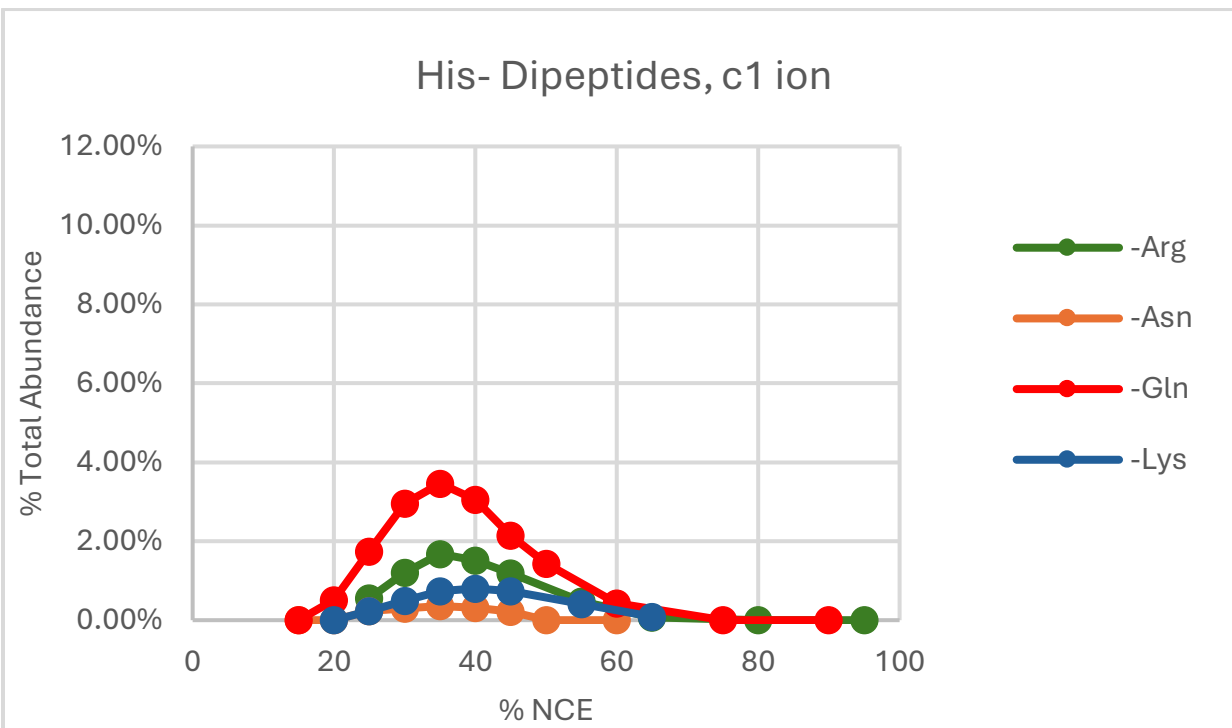
His- Dipeptides, c1 ion
% Total Abundance
12.00%
10.00%
8.00%
6.00%
4.00%
2.00%
0.00%
0
20
40
60
80
100
% NCE
-Arg
-Asn
-Gln
-Lys

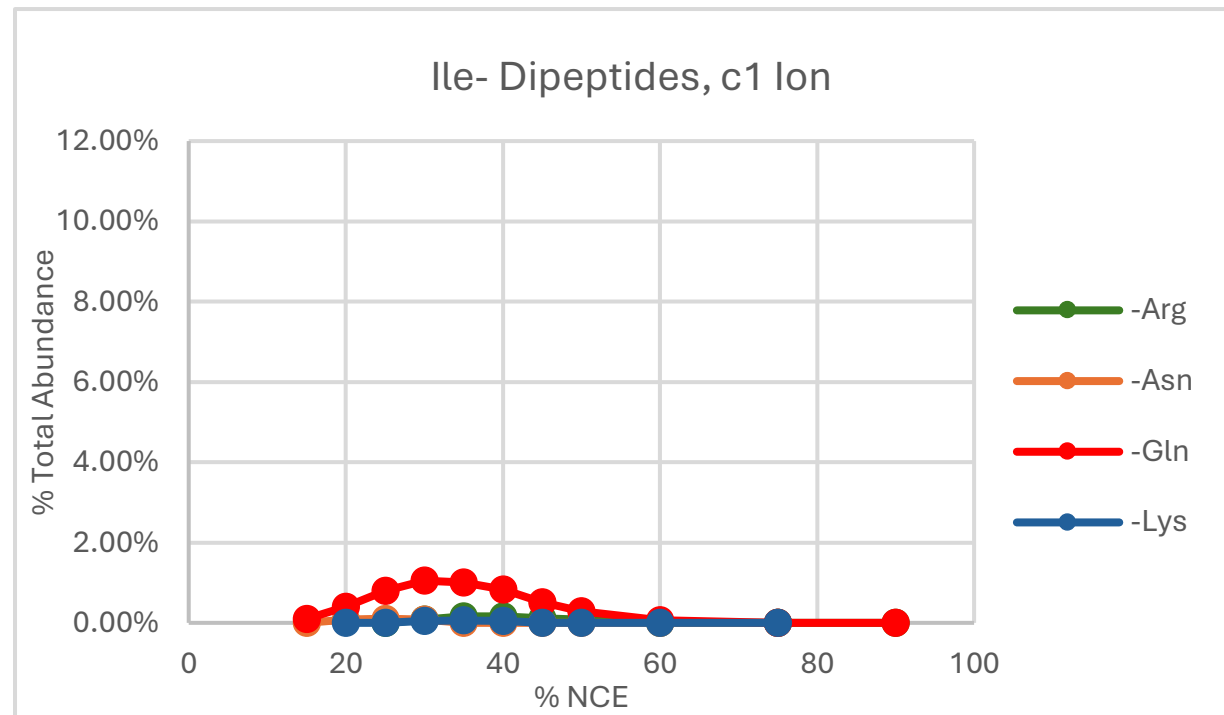
Ile- Dipeptides, c1 Ion
% Total Abundance
12.00%
10.00%
8.00%
6.00%
4.00%
2.00%
0.00%
0
20
40
60
80
100
% NCE
-Arg
-Asn
-Gln
-Lys

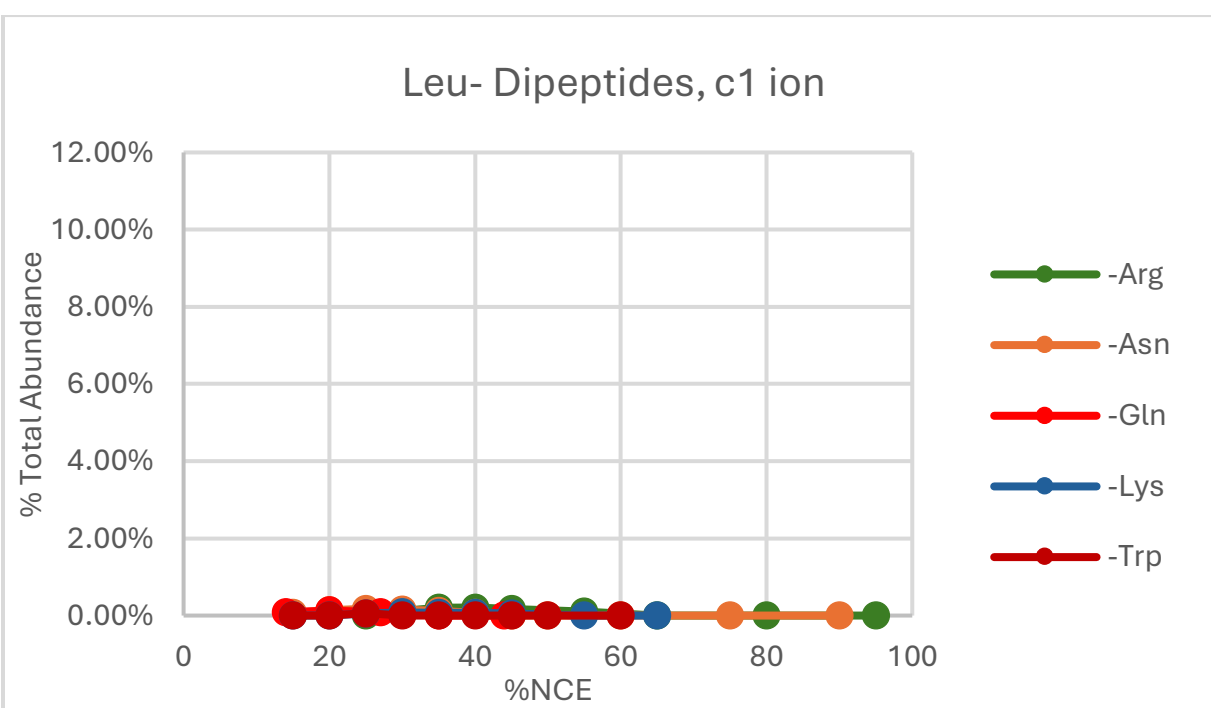
Leu- Dipeptides, c1 ion
% Total Abundance
12.00%
10.00%
8.00%
6.00%
4.00%
2.00%
0.00%
0
20
40
60
80
100
%NCE
-Arg
-Asn
-Gln
-Lys
-Trp

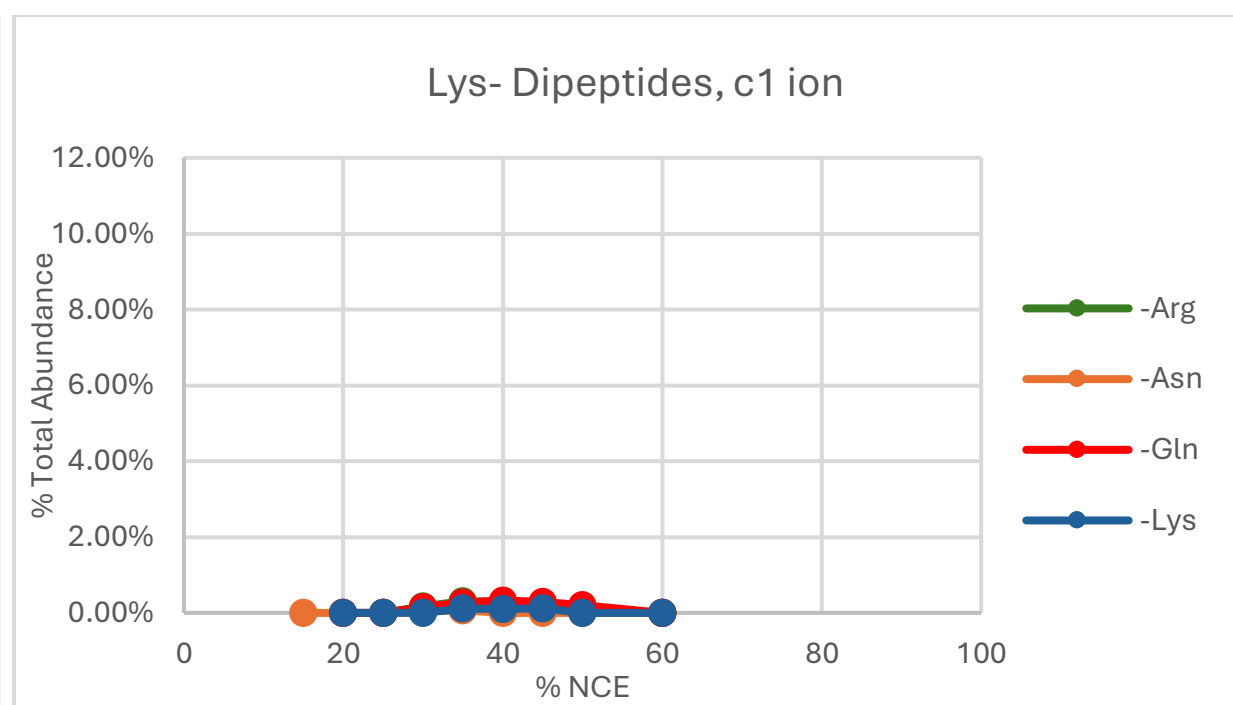
Lys- Dipeptides, c1 ion
% Total Abundance
12.00%
10.00%
8.00%
6.00%
4.00%
2.00%
0.00%
0
20
40
60
80
100
% NCE
-Arg
-Asn
-Gln
-Lys

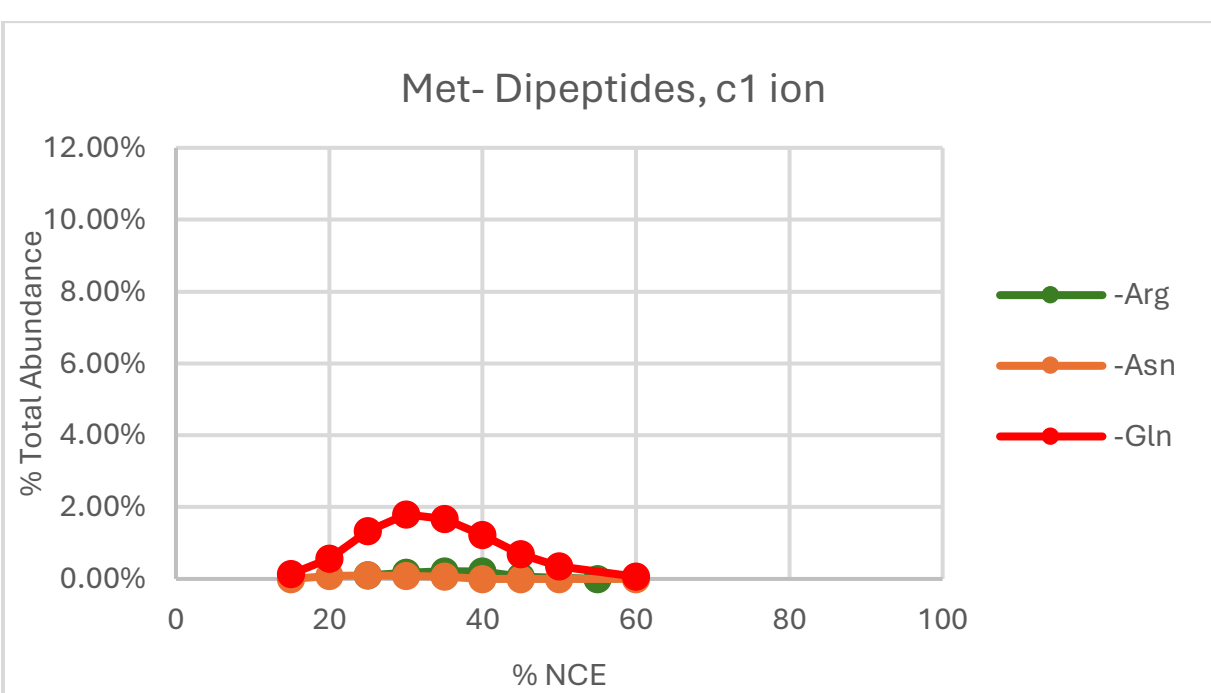
Met- Dipeptides, c1 ion
% Total Abundance
12.00%
10.00%
8.00%
6.00%
4.00%
2.00%
0.00%
0
20
40
60
80
100
% NCE
-Arg
-Asn
-Gln

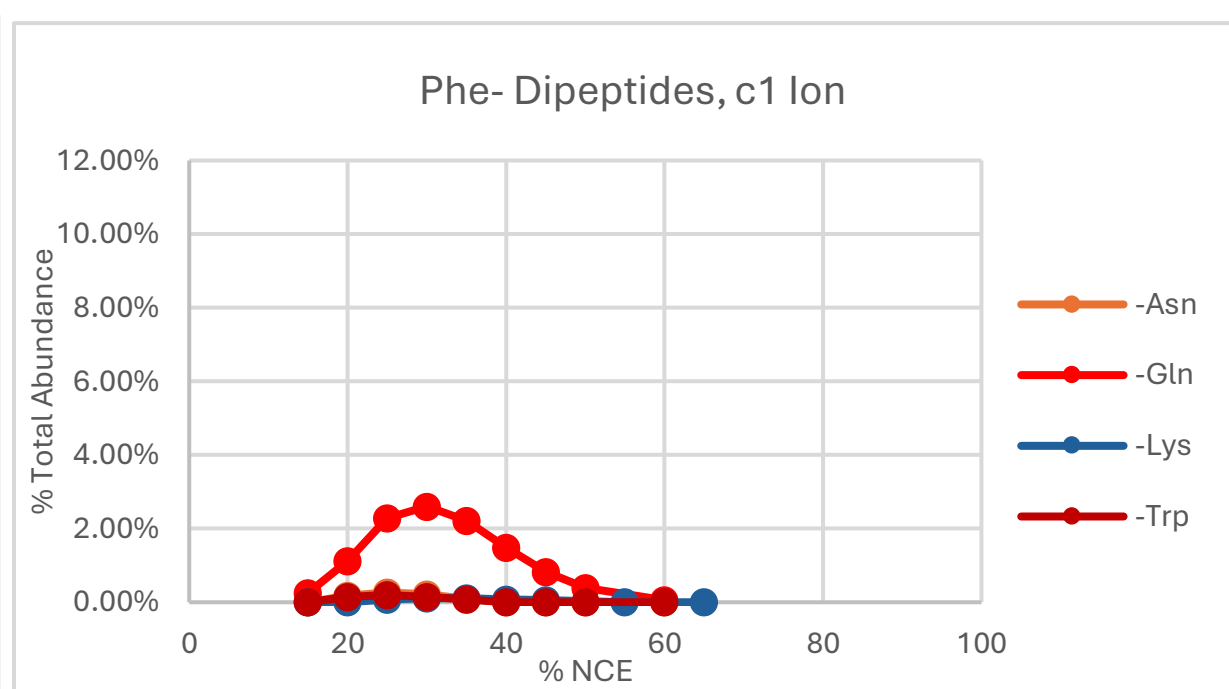
Phe- Dipeptides, c1 Ion
% Total Abundance
12.00%
10.00%
8.00%
6.00%
4.00%
2.00%
0.00%
0
20
40
60
80
100
% NCE
-Asn
-Gln
-Lys
-Trp

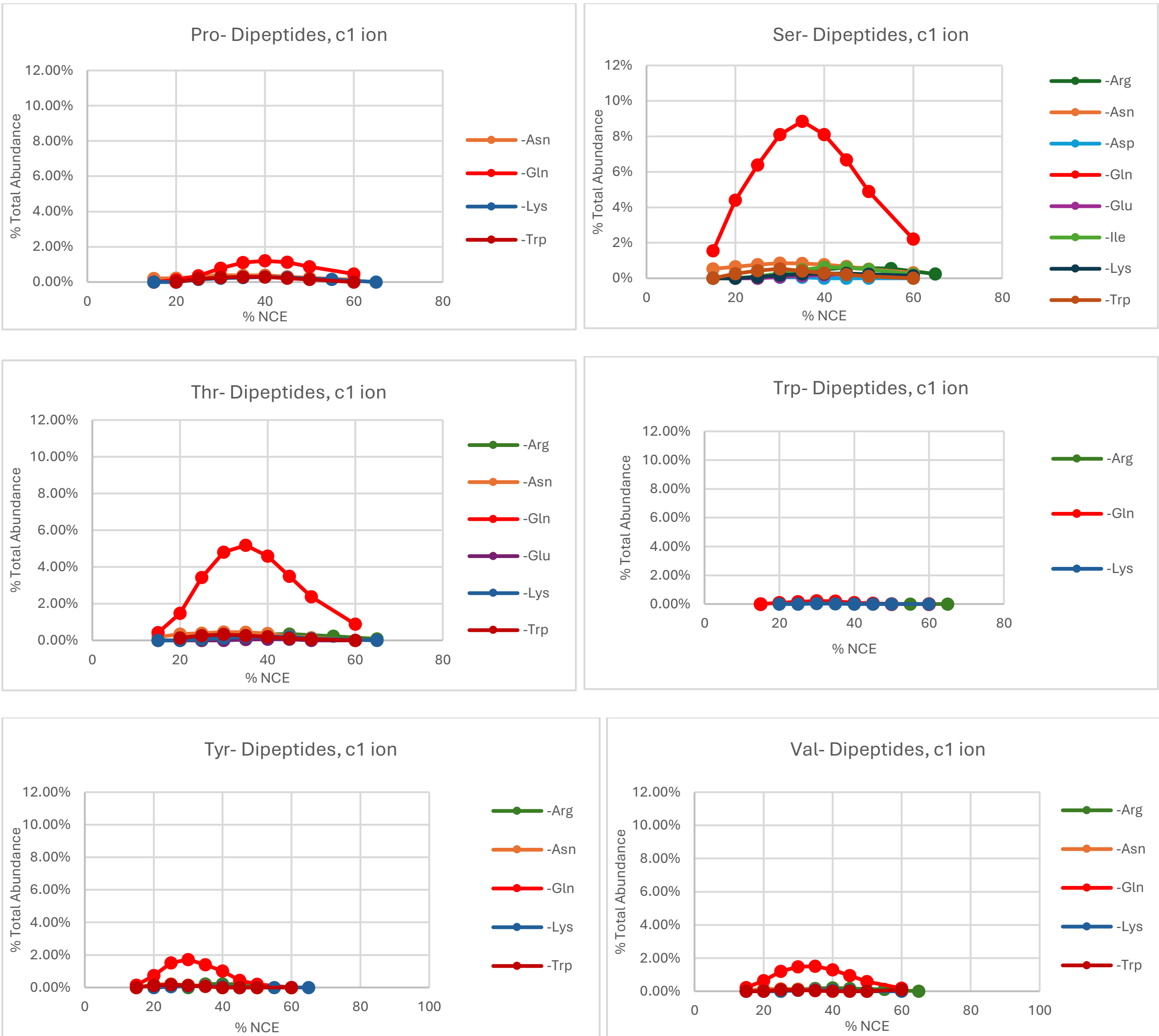

Supporting Information Figure S2. Comparison of c_1 ion relative abundance, where each pane contains dipeptides that share the same N-terminal amino acid. Here, abundance values and total ion abundance were extracted from the NIST Tandem mass spectral library.

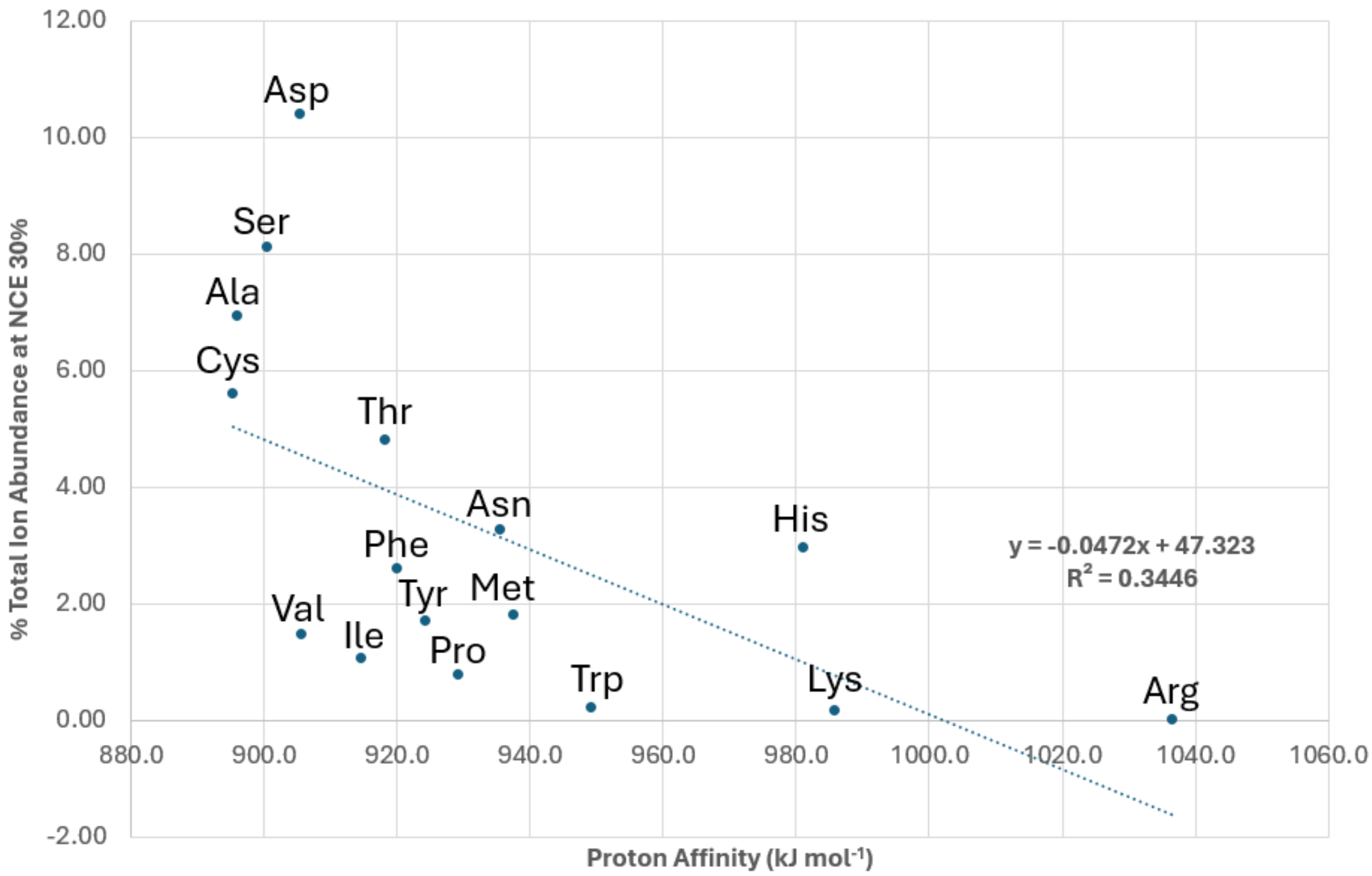

Supporting Information Figure S3. Correlation between c1 ion relative abundance and proton affinity, for which the values are listed in Table 1.

Supporting Information Table S1. Chemical formula and corresponding monoisotopic m/z values for c_1 ions for each amino acid.

Amino Acid	c_1 Ion Chemical Formula	c_1 Ion Mono. m/z
**Ala**	C3 H9 N2 O1	89.0709
**Arg**	C6 H16 N5 O1	174.1349
**Asn**	C4 H10 N3 O2	132.0768
**Asp**	C4 H9 N2 O3	113.0608
**Cys**	C3 H9 N2 O1 S1	121.0430
**Cys(CAM)**	C5 H12 N3 O2 S1	178.0645
**Glu**	C5 H11 N2 O3	147.0764
**Gln**	C5 H12 N3 O2	146.0924
**Gly**	C2 H6 N1 O1	75.0553
**His**	C6 H11 N4 O1	155.0927
**Ile**	C6 H15 N2 O1	131.1179
**Leu**	C6 H15 N2 O1	131.1179
**Lys**	C6 H16 N3 O1	146.1288
**Met**	C5 H13 N2 O1 S1	149.0743
**Phe**	C9 H13 N2 O1	165.1022
**Pro**	C5 H11 N2 O1	115.0866
**Ser**	C3 H9 N2 O2	105.0659
**Thr**	C4 H11 N2 O2	119.0815
**Trp**	C11 H14 N3 O1	204.1131
**Tyr**	C9 H13 N2 O2	181.0972
**Val**	C5 H13 N2 O1	117.1022

(1) Grzetic, J.; Oomens, J. Spectroscopic Identification of Cyclic Imide b2-Ions from Peptides Containing Gln and Asn Residues. *Journal of the American Society for Mass Spectrometry* **2013**, *24* (8), 1228-1241. DOI: 10.1007/s13361-013-0661-6.
